# Review of Biosimilar Trials and Data on Adalimumab in Rheumatoid Arthritis

**DOI:** 10.1007/s11926-018-0769-6

**Published:** 2018-08-09

**Authors:** Sizheng Zhao, Laura Chadwick, Eduardo Mysler, Robert J. Moots

**Affiliations:** 1 Department of Musculoskeletal Biology I, Clinical Sciences Centre, Institute of Ageing and Chronic Disease, University of Liverpool, Aintree University Hospital, Longmoor Lane, Liverpool, L9 7AL UK; 2 Organización Medica de Investigación, Buenos Aires, Argentina

**Keywords:** Adalimumab, Biosimilar, Rheumatoid arthritis, Amgevita, Cyltezo, Imraldi

## Abstract

**Purpose of Review:**

Adalimumab is one of the top-selling drugs worldwide. Its imminent patent expiration has seen the emergence of numerous biosimilar agents. In this article, we recap the evidence from bio-originator trials in rheumatoid arthritis (RA) to provide context for a critical review of biosimilar trial data.

**Recent Findings:**

Currently, three adalimumab biosimilars are approved in Europe and/or the USA: Amgen’s ABP 501 (AMJEVITA/Solymbic), Boehringer Ingelheim’s BI 695501 (Cyltezo) and Samsung Bioepis’s SB5 (Imraldi). All three agents met their pre-specified equivalence criteria. Subtle differences in adverse events and clinical responses between the reference and biosimilar products were noted.

**Summary:**

The introduction of adalimumab biosimilars will offer exciting opportunities in improving treatment access and increasing treatment options for RA and other licensed indications. Real-world data will further provide assurances on efficacy as well as safety.

## Introduction

Rheumatoid arthritis (RA) is a chronic, autoimmune, systemic inflammatory disease. If inadequately treated, it can lead to irreversible joint damage and disability at significant costs to the individual as well as the wider economy. Life expectancy of patients with severe RA is reduced by 10 years on average [1–3], whilst the total costs of RA to society were estimated at €45 billion in Europe and $52 billion in the USA in 2006 [4].

With improved understanding of RA’s molecular pathophysiology, increasingly targeted treatments were developed in addition to conventional synthetic disease-modifying antirheumatic drugs (csDMARDs) such as methotrexate (MTX). Around the turn of the century, biologic drugs were introduced that selectively targeted tumour necrosis factor (TNF). TNF inhibitors (TNFi), along with other biologic DMARDs (bDMARDs), formed an effective second-line for those with inadequate response to csDMARDs and dramatically improved mortality and outcomes for RA patients [5–7].

In 2002, Humira, the originator adalimumab, became the third TNFi to be approved in the USA after infliximab and etanercept. It is the best-selling drug worldwide, with global sales of $18 billion in 2017 alone [8]. It is also one of the most versatile, being additionally approved for use in ankylosing spondylitis/axial spondyloarthritis, plaque psoriasis, psoriatic arthritis, Crohn’s disease, ulcerative colitis, polyarticular juvenile idiopathic arthritis, hidradenitis suppurativa and non-infectious uveitis [9]. It has become the benchmark agent against which newer biologic or targeted synthetic DMARDs are compared.

The inevitable patent expiration for TNFi stimulated programmes to develop molecules that, whilst not identical to the originator, could be considered to be a biological equivalent of a ‘generic’. Such ‘biosimilars’ are defined as biological agents that are similar in terms of quality, safety and efficacy to the licensed reference product (RP) [10]. Biosimilars of infliximab and etanercept are already established worldwide and, for most countries, biosimilar adalimumab will enter the market in the last quarter of 2018 when Humira’s exclusivity expires.

In this article, we summarise the pharmacology and clinical efficacy of adalimumab to provide context for a critical review of evidence from randomised controlled trials of its biosimilars in RA. We focus on biosimilars that are approved by the European Medicines Agency (EMA) and/or the US Food and Drugs Administration (FDA).

## Pharmacology

**Fig. 1:**
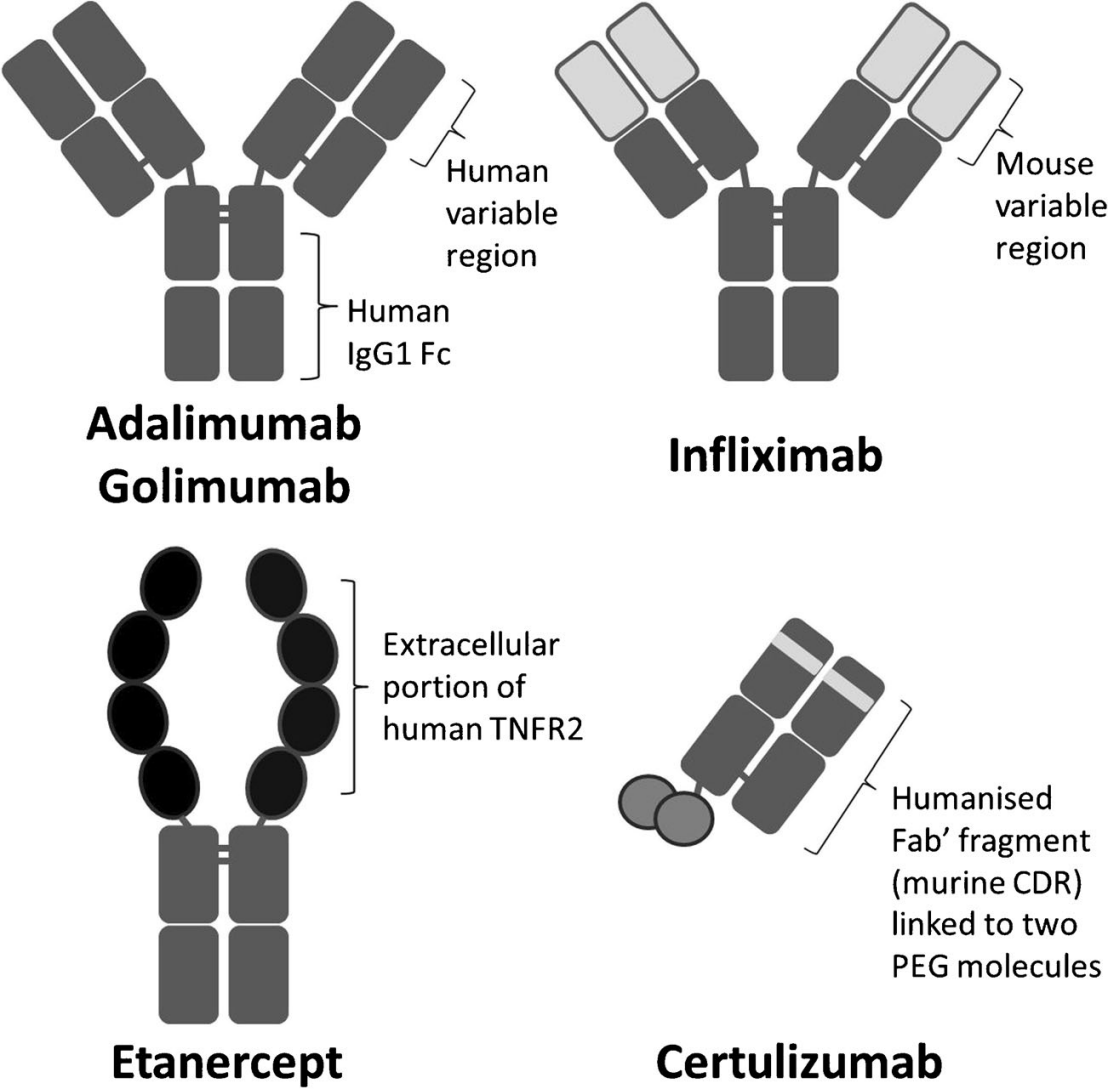
Adalimumab structure compared with other TNF inhibitors. TNFR2 TNF receptor 2, Fc fragment crystallisable region, Fab’ antigen-binding fragment, CDR complementarity-determining regions, PEG polyethylene glycol

Adalimumab is a recombinant, fully human, IgG1 monoclonal antibody that is structurally and functionally indistinguishable from naturally occurring human IgG1 (Fig. 1). It was engineered through phage display technology and is produced in a Chinese hamster ovary cell line [11].

Adalimumab is administered by subcutaneous injection, reaching peak serum concentrations after approximately 131 h [12]. It is widely distributed, including into the synovium. Similar to naturally occurring human IgG1, its elimination half-life is around 10 to 14 days. Adalimumab binds specifically to TNF-alpha (both soluble and membrane-bound) and blocks their interaction with p55 and p75 cell surface TNF receptors [12].

Despite being a fully human antibody, up to 30% of RA patients develop anti-drug antibodies (ADAb) to adalimumab [13]. ADAb can block the drug from binding to its target and/or form immune complexes; they have been shown to decrease serum drug levels and increase markers of inflammation in RA patients [13].

## Clinical Efficacy of Adalimumab

The efficacy of adalimumab in both early and long-standing RA has been demonstrated by several phase III randomised controlled trials (Table 1) and open-label extensions [14, 15]. For the remainder of the review, we refer to the standard dosing of 40 mg every 2 weeks unless otherwise stated. Common outcome measures included ACR responses (20, 50 or 70% improvement in the ACR core set measurements), DAS28 remission (< 2.6), EULAR response (based on changes in DAS28) and patient-reported physical disability assessed via Health Assessment Questionnaires (HAQ). Radiographic progression was measured by modified or original total Sharp score.

**Table 1:** Study and patient characteristics of adalimumab trials in rheumatoid arthritis

Study	Trial size and duration	Treatment arms	Country	Treatment history	Age, years	Females, %	Disease duration, years	Mean TJC, n	Mean SJC, *n*	HAQ
Weinblatt 2003 (ARMADA)	*n* = 271 24 weeks	Humira 20 mg + MTX Humira 40 mg + MTX Humira 80 mg + MTX Placebo + MTX	35 sites in the USA and Canada	On stable MTX only, failed at least 1 DMARD, biologic naive	55.5	77	12.3	28.9	17.2	1.56
Keystone 2004	*n* = 619 52 weeks	Humira 20 mg + MTX Humira 40 mg + MTX Placebo + MTX	89 sites in the USA and Canada	On stable MTX only, biologic naive	56.5	75	10.9	27.8	19.3	1.48
Breedveld 2006 (PREMIER)	*n* = 799 2 years	Humira 40 mg + MTX Humira 40 mg + placebo Placebo + MTX	11 sites in Australia, 85 Europe, 37 N. America	MTX naive	52.0	74	0.7	31.6	21.7	1.5
Furst 2003 (STAR)	*n* = 636 24 weeks	Humira 40 mg + standard DMARD Placebo + standard DMARD	69 sites in the USA and Canada	Stable standard treatment (DMARD, low-dose steroids, NSAIDs, alnalgesia), biologic naive	55.4	79	10.4	27.5	21.1	1.40
van de Putte 2004	*n* = 544 26 weeks	Humira 20 mg weekly Humira 20 mg alternate weeks Humira 40 mg weekly Humira 40 mg alternate weeks Placebo	52 sites Europe, Canada, Australia	Failed at least one DMARD, biologic naive	53	77	11	34.4	19.8	1.9
Cohen 2016	*n* = 526 26 weeks	ABP 501 + MTX Humira + MTX	40 sites in E. Europe, 12 W. Europe, 38 N. America. 2 Mexico	Stable MTX, adalimumab naive, no more than 1 previous biologic	55.9	81	9.4	24.1	14.4	1.49
Weinblatt 2018	*n* = 544 52 weeks	SB5 + MTX (rerandomised at w24) Humira + MTX	51 sites in E. Europe and Republic of Korea	Stable MTX, biologic naive	51.2	81	5.5	24.0	15.6	1.4
Cohen 2018	*n* = 645 58 weeks	BI 695501 + MTX (rerandomised at w24) Humira + MTX	115 sites (68% E. Europe, 4% W. Europe, 19% USA, 8% Chile, 2% Asia)	Stable MTX, adalimumab naïve, no more than 1 previous biologic	53.7	83	7.2	25.1	16.5	1.5 (median)
Jani 2016	*n* = 120 12 weeks	ZRC3197 + MTX Humira + MTX	11 sites in India	Stable MTX, biologic naive	45	83	3.7	17.0	11.8	1.7
Jamshidi 2017	*n* = 136 24 weeks	CinnoRA + MTX Humira + MTX	10 sites in Iran	Inadequate response to csDMARDs	47.9	87	–	9.6	9.7	1.32
Alten/Genovese 2017 (abstract)	*n* = 728 24 weeks	FKB327 + MTX Humira + MTX	105 sites in E. and W. Europe, N. and S. America.	Stable MTX, adalimumab naïve	55.3	–	8.5	–	–	–

*MTX* methotrexate, *SJC* swollen joint count, *TJC* tender joint count, *HAQ* Health Assessment Questionnaire

As monotherapy, adalimumab recipients had significantly better outcomes than placebo in terms of function, ACR and EULAR responses [16]. The exposure-time-adjusted incidence of adverse events (AEs) was comparable to placebo, including serious infections and malignancies. However, as biosimilar trials did not include monotherapy, it will not be discussed further in this review.

Adalimumab monotherapy was not superior to MTX monotherapy in terms of ACR response or DAS28 remission over 2 years in the PREMIER study (of MTX naive, early RA patients) [17]. It was, however, superior to MTX monotherapy at improving function and slowing/preventing radiographic progression. Both monotherapy groups had comparable incidences of AEs, including serious infections.

Adalimumab combination therapy with MTX was superior to adalimumab or MTX monotherapies for all clinical and radiographic outcomes in early as well as established disease [17–19]. ACR responses were superior when standard csDMARD treatment was combined with adalimumab than without [20]. Incidence of AEs was similar in combination and monotherapy groups [17–20]. Adalimumab combination therapy with MTX was associated with higher incidence of serious infections than MTX monotherapy [17, 19].

Other bDMARDs were similar in efficacy to adalimumab in head-to-head trials [21–24], network meta-analysis [25] and observational cohorts [26, 27]. Only IL6 inhibitors (as monotherapies) [28, 29] and the targeted synthetic DMARD, Baricitinib (as combination therapy), demonstrated superiority [30].

## Adalimumab Biosimilars

Biosimilar approval does not require the manufacturer to re-establish efficacy but is instead based on the demonstration that there are no clinically meaningful differences from the RP [10, 31, 32]. Once biosimilarity has been established in one indication, the drug may be approved for additional indications held by the RP without comparative clinical trials. Extrapolation of indication reduces the number and size of clinical trials required, thus decreasing financial cost and, potentially, increasing access [32].

At the time of writing, three adalimumab biosimilars are approved in the EU and/or the USA: Amgen’s ABP 501, Boehringer Ingelheim’s BI 695501 and Samsung Bioepis’s SB5. In the following sections, we will review each in terms of clinical outcomes and trial characteristics. Other biosimilars will be mentioned in passing, including FKB327 by Fujifilm Kyowa Kirin Biologics which has phase III RA trial published in abstract form [33, 34]; Sandoz’s GP2017 which has phase III plaque psoriasis trial in abstract form [35] and has been accepted for regulatory review [36]; and CinnoRA (Iran) and Exemptia (India) which have published RCTs on PubMed [37, 38]. Additional pipeline biosimilars with completed/ongoing phase III trials are listed in Table 2. Torrent Pharmaceuticals (India) was the second company in the world to launch an adalimumab biosimilar, Adfrar [39]. However, its trial evidence is not PubMed accessible and will not be included in this review.

**Table 2:** Adalimumab biosimilar pipeline as of April 2018

Adalimumab biosimilar	Manufacturer	Trial status	Trial registration number
FKB327	Fujifilm Kyowa Kirin Biologics	Phase III completed	NCT02260791
PF-06410293	Pfizer	Phase III completed	NCT02480153
CHS-1420	Coherus BioSciences	Phase III completed	NCT02489227
M923 / BAX923	Momenta	Phase III (PsO) completed	NCT02581345
MYL-1401A	Mylan	Phase III (PsO) completed	NCT02714322
LBAL	LG Life Sciences	Phase III ongoing	NCT02746380
MSB11022	Merck KGaA	Phase III ongoing	NCT03052322
GP2017	Sandoz	Phase III ongoing	EudraCT: 2015-003433-10
BCD-057	Biocad	Phase III (PsO) ongoing	NCT02762955
ONS-3010	Oncobiologics	Phase III (PsO) ongoing	EudraCT: 2015-004614-26

*PsO* plaque psoriasis

## ABP 501

Amgen’s ABP 501 was the first adalimumab biosimilar to be approved by the FDA in 2016 (as AMJEVITA) and by the EMA in 2017 (as AMGEVITA/Solymbic). The manufacturer demonstrated that ABP 501 and the RP were highly similar in structure, function and pharmacokinetics (PK) [40–42]. Subsequent phase III studies were conducted in both plaque psoriasis [43] and RA [44•].

The RA trial was a randomised, double-blind, active comparator-controlled, 26-week equivalence study, comparing ABP 501 with US- and EU-sourced RP [44•]. Participants did not undergo switching between ABP 501 and the RP. The trial included 526 patients with moderate to severe active RA despite MTX. Unlike the original RP trials, 28% of patients had prior exposure to bDMARD (but not adalimumab); whether these participants failed prior bDMARDs due to inefficacy or AEs was not discussed.

The primary endpoint was the risk ratio (RR) of achieving ACR20 at week 24, which was achieved in 74.6% of the ABP 501 group and 72.4% in the RP group (using Full Analysis Set (FAS))—numerically higher than response rates from pivotal RP trials. Results of the per-protocol set (PPS) analysis were similar (raw results were not provided). The ACR20 RR was 1.039 (90%CI 0.954 to 1.133), which was within the margin of 0.738 to 1.355 required to demonstrate equivalence. Equivalence was also demonstrated in patients with plaque psoriasis as monotherapy [43].

In the RA study, the RR of ACR20 for ABP 501 was 1.421 (95% CI 1.086 to 1.860) after 2 weeks, suggesting superiority over the RP [45, 46]. This was also seen at week 12. However, the difference was not statistically significant at other time points. Furthermore, there were no significant differences in secondary outcomes (ACR50/70, DAS28). The EMA therefore concluded that differences were likely a chance finding rather than superiority in onset of action.

The proportions of binding ADAb (38.3 vs 38.2%) and neutralising ADAb (9.1 vs 11.1%) were similar for ABP 501 and the RP, respectively. More patients discontinued treatment (6.8 vs 4.6%) and the study (8.0 vs 4.2%) in the ABP 501 group; there were no specific AEs leading to withdrawal. Liver enzyme elevations occurred in 4.9% of the ABP 501 group and in 3.8% in the RP group. This signal for ABP 501 was also seen in the psoriasis study (5.9 vs 2.5%) [43]. However, the EMA concluded that these events occurred to a large extent in subjects with abnormal baseline values [45, 46].

Injection site reactions were less common in the ABP 501 group (1.7 vs 5.2%). This was also seen in the psoriasis trial (1.3% v 3.8%) and explained as differences in the excipients rather than biosimilarity.

## BI 695501

Boehringer Ingelheim’s BI 695501 (Cyltezo) was approved by the EMA and FDA in 2017. It was shown to be similar to the RP in structure, function and PK [47, 48•]. Equivalence studies were published in RA [48•], completed in plaque psoriasis (NCT02850965) and is ongoing in Crohn’s disease (NCT02871635).

VOLTAIRE-RA was a randomised, double-blind, parallel-arm, 58-week study, comparing BI 695501 with US-sourced RP [48•]. The trial cohort comprised 645 patients with moderate to severe RA despite MTX, 27% of whom had prior exposure to bDMARD (but not adalimumab); again, reasons for prior bDMARD failure were not provided. At week 24, patients on the RP were rerandomised to BI 695501 or to stay on the RP. At week 48, all patients were entered into an open-label extension until week 58.

Co-primary endpoints were percentage of patients achieving ACR20 at 12 and 24 weeks (using FAS). At week 12, 67.0% of BI 695501 recipients and 61.1% of RP recipients achieved ACR20. The difference (5.9%; 90%CI − 0.9 to 12.7) was within − 12 to 15% set by the FDA to demonstrate equivalence; PPS results were similar (4.3%; 95%CI − 2.8 to 11.3). At week 24, 69.0 and 64.5% of BI 695501 and RP recipients achieved ACR20, respectively. This difference of 4.5% (90%CI − 3.4 to 12.5) was within ± 15% set by the EMA (PPS 1.6%; 95%CI − 5.3 to 8.5). Secondary endpoints (ACR and EULAR responses, DAS28, SF-36) were also similar.

There was a trend for superior ACR20 response for BI 695501 [49]. At week 4 (the most steep and therefore sensitive part of the dose-response curve), 6.9% more of the BI 695501 group reached ACR20 than the RP group (confidence interval not provided). Similar trends were seen at weeks 4 and 12. However, differences between the two groups were smaller for ACR50 and ACR70 and did not persist over time. Another discrepancy was noted regarding persistence of ACR20 response: 74.3% of the BI 695501 group achieved ACR20 at week 24 but only 67.9% at week 48, while an increase was seen in the RP group (66.8 and 70.2%, respectively). Patients who switched from the RP to BI 695501 also showed trends of falling ACR 20 response (72.3% at week 24, 64% at week 48). This was dismissed as likely a chance finding since it was not seen with other outcome measures.

There were no clinically meaningful differences in proportions of ADAb (BI 695501 47.4%, RP 53.0% up to week 24); neutralising ADAb prevalence was also similar (raw results were not provided). The number of patients with at least one AE was 59.6% in the BI 695501 group and 60.0% in the RP group. Several safety outcomes were in favour of BI 695501: serious AEs (5.6 vs. 9.7%), injection site reactions (1.9 vs 5.7%), serious infections (0.6 vs 4.0%) and hypersensitivity reactions (2.8 vs 4.6%). However, three aspects of safety initially raised the concern about the safety profile of BI 695501. First, haematological disorders were more frequent in the continuous BI 695501 group than continuous RP group (5.2 vs 2.9%): anaemia (8 patients, 2.5%) and ‘haemoglobin decreased’ (2 patients, 0.6%) were reported exclusively in the BI 695501 group. These AEs were mostly mild to moderate and did not lead to drug discontinuation. Furthermore, five of these patients had low haemoglobin at baseline. Second, bone fractures occurred exclusively in the BI 695501 group (7 patients, 2.2%). This was also observed in the PK trial [47]. However, the incidence rate (20.8 per 1000 patient-years) was within the range of bone fracture risk in the general population (8.5 to 36.0 per 1000 patient-years). Third, patients who received BI 695501 more frequently screened positive for TB (no active cases) at week 48: 8 patients (2.8%) in the BI 695501 group, 1 patient (0.7%) in the RP group and 8 patients (5.7%) in the switch group (RP to BI 695501). All patients screened negative for TB at the start of the trial. The EMA accepted that all three were rare events and that the minor differences were likely a chance finding.

## SB5

Samsung Bioepis’s SB5 (Imraldi) was approved by the EMA in 2017. Again, it was similar to the RP in structure, function and PK [50, 51•]. Its phase III study was a randomised, double-blind, parallel-arm, 52-week trial of 544 patients with moderate to severe RA despite MTX, comparing SB5 with US- and EU-sourced RP. Unlike the two biosimilar trials discussed above, all patients in this study were biologic naive at baseline. At week 24, patients on the RP were rerandomised to SB5 or the RP until week 52.

The primary endpoint was ACR20 response rate at week 24 using PPS, which was achieved by 72.4% of the SB5 and 72.2% of the RP group. The difference of 0.1% (95%CI − 7.8 to 8.1) was within ± 15% required to demonstrate equivalence. Results using FAS were similar (68.0 vs 67.4%, respectively) with a difference of 0.8% (95%CI - 7.0 to 8.6).

ACR20 and ACR50 at week 52 showed a trend of superior response in both SB5 and SB5/RP-switch groups compared to the RP group, using the PPS (upper limit of CI: 15.9%, exceeding ± 15%) but not using FAS [52]. The percentage of subjects who achieved major clinical response (ACR70 for 6 consecutive months) at week 52 was 15.7% in the SB5, 15.3% in the switch group and 9.7% in the RP group. Since the study was not designed to show similarity at week 52, these findings were not considered as superior response. Other response measures (ACR50/70, DAS28) were similar.

The incidence of ADAb was similar between SB5 and RP groups (33.1 vs 32.0% up to week 24, respectively). Overall, treatment emergent AEs occurred in 35.8% of the SB5 and 40.7% of the RP group; 10.1 and 11.7%, respectively, were considered related to the study drug. The number of injection site reactions was higher in RP recipients (32 reactions in 4 patients) compared to SB5 recipients (9 reactions in 8 patients) at week 52, although the proportions of patients were comparable (3.1 vs 3.0%, respectively). The rate of discontinuation due to AEs was lower in the SB5 than RP group (0.7 vs 3.3% at week 24; 1.5 vs 2.4% at week 52).

## Equivalence and Switching

An interesting observation in biosimilar trials was that clinical responses in their RP groups were often different from responses reported in pivotal RP trials. For example in the EGALITY plaque psoriasis study, PASI75 response (75% improvement in Psoriasis Area and Severity Index) of the etanercept RP group (76%) was much higher than in previous RP studies (47 to 49%) [53–55]. Similarly, PASI75 response at week 16 to the RP was 86% in the ABP 501 trial but 71 to 80% in pivotal trials [43, 56, 57]. Similar trends have also been observed for etanercept and infliximab trials in RA [58]. These discrepancies may be attributable to fundamental differences in study design and/or characteristics of the study population.

**Fig. 2:**
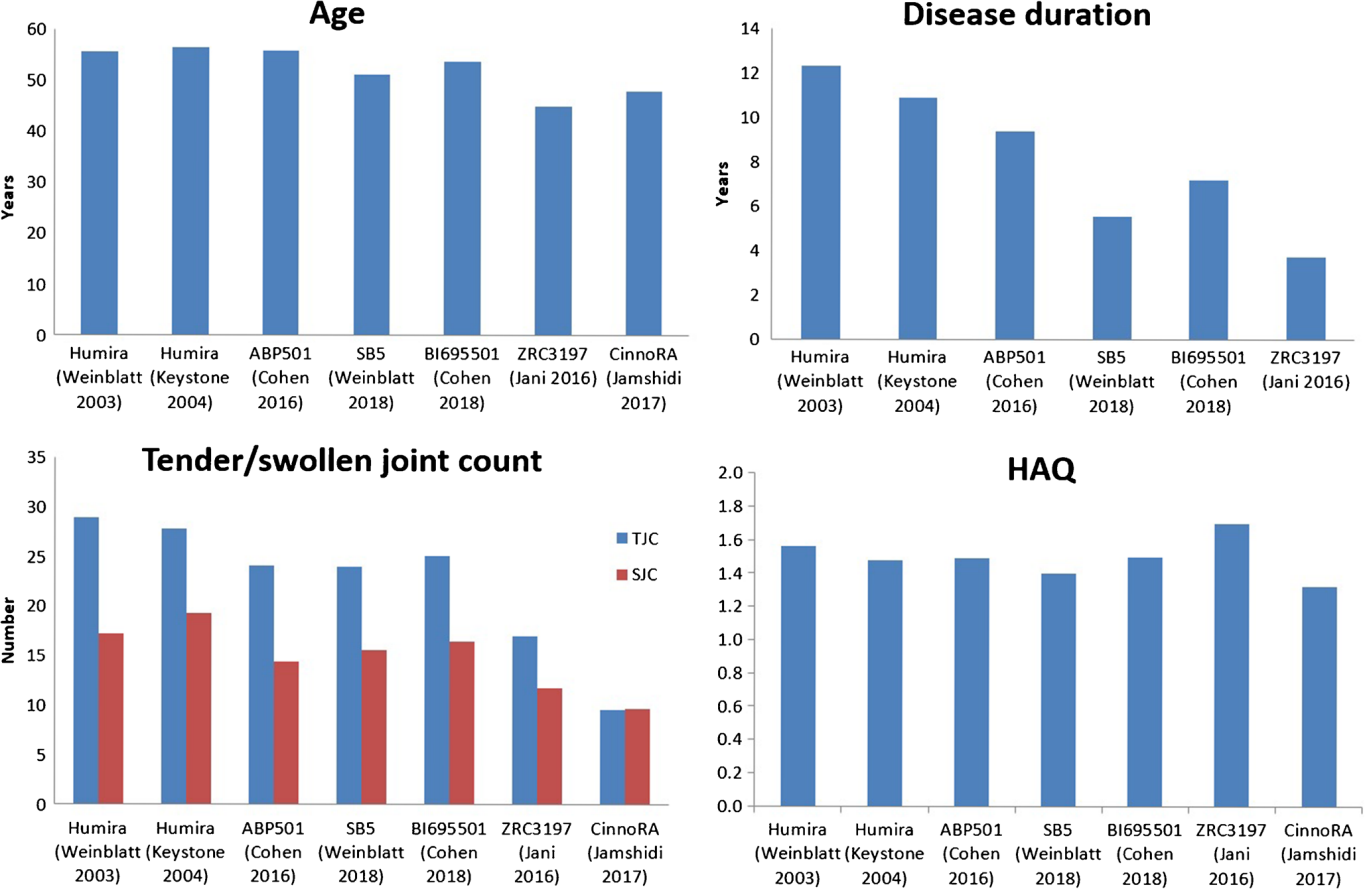
Differences in patient characteristics between pivotal and biosimilar trials of adalimumab

**Fig. 3:**
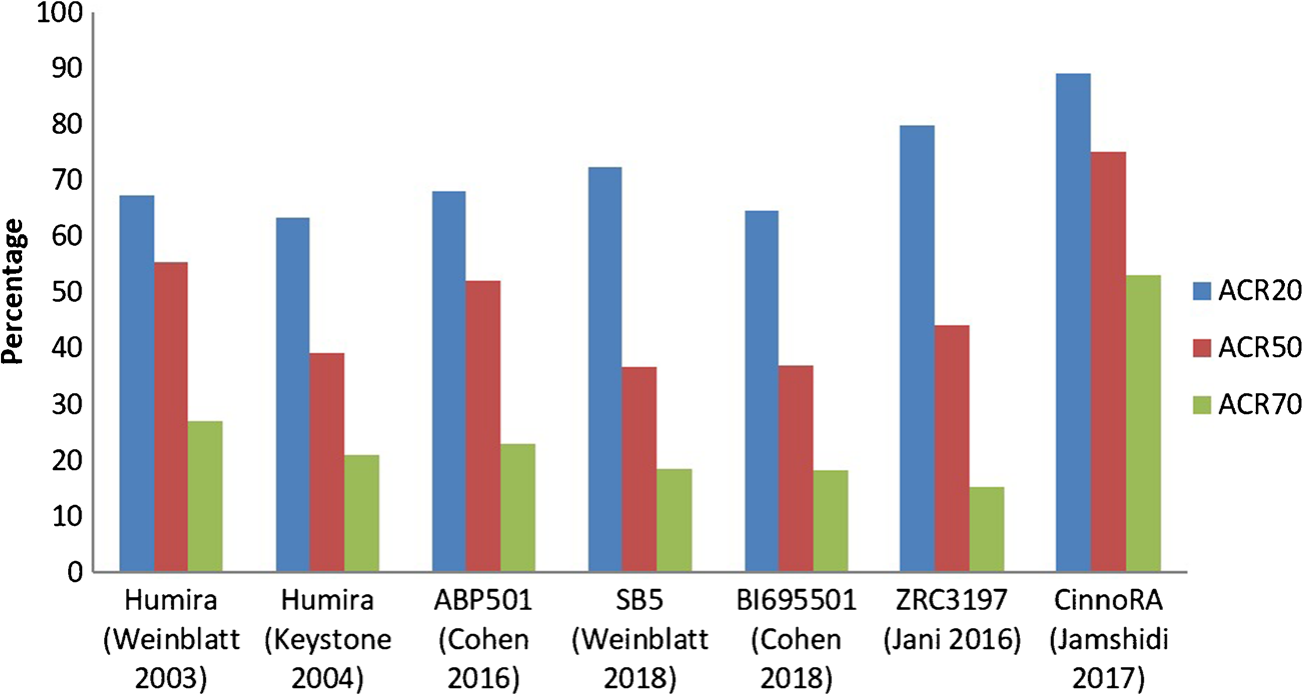
Differences in ACR response rates for the reference product in pivotal and biosimilar trials

Unlike the pivotal RP trials that were mostly conducted in North America, trials for SB5, ABP 501 and BI 695501 were predominantly conducted in Eastern European countries (Table 1). These patients may receive better healthcare if enrolled, thereby incentivising trial engagement but possibly introducing bias. It is also possible that the greater response rates were due to these patients having less severe disease. The two Asian biosimilar trials both reported less severe disease at baseline; their RP group also had greater response than the pivotal RP trials (Fig. 2). These differences were less evident for the three EMA/FDA-approved adalimumab biosimilars. However, it is worth noting that patients in the ABP 501 and BI 695501 trials were not all biologic naive and yet had comparable response rates to the biologic naive cohorts of pivotal trials (Fig. 3); response to a second TNFi is recognised to be poorer [59, 60]. Unfortunately, neither studies stated whether participants stopped previous bDMARDs due to inefficacy or AEs. There were also subtle differences in participant age, disease duration and HAQ (Fig. 2).

These subtle differences in trial characteristics, as well as minor discrepancies in rare safety signals, should be considered when drawing conclusions on the true biosimilarity of these agents. This has implications for switching between the RP and biosimilars. Unlike biosimilar infliximab and etanercept [61•], there are not many open label extension or pharmacovigilance studies for biosimilar adalimumab. In the SB5 trial, patients receiving the RP (*n* = 273) were rerandomised to switch to SB5 (*n* = 125) or to continue with the RP (*n* = 129) at week 24, until the end of the study at week 52. Switching had no impact on efficacy, safety or immunogenicity [62]. An open -abel extension to 72 weeks has been published in abstract form and reports no additional concerns [63, 64]. In the BI 695501 trial, patients receiving the RP (*n* = 321) were rerandomised at week 24 to switch to BI 695501 (*n* = 147) or remain on the RP (*n* = 148) until week 42 [48•]. A small subgroup of patients was followed up until week 58 to assess safety. Again, no differences were reported for efficacy, safety or immunogenicity.

However, these RCTs were not powered to show the significance of differences in rare AEs. In addition, trials have stringent inclusion and exclusion criteria that may not reflect real-world patients, such as those with comorbidities. Therefore, pharmacovigilance studies are essential to gather clinical evidence of the benefits and risks of switching in all patients. We refer the reader to reference [61•] for a more detailed discussion on biosimilar switching.

## Conclusion

The year 2018 will see several biosimilars of adalimumab emerge in clinical practices worldwide. The three EMA/FDA-approved agents discussed in this review each have robust RCT evidence that meet pre-defined equivalence criteria, although subtle differences in clinical responses and AEs between the reference and biosimilar products were noted. As with TNFi biosimilars already on the market, real-world data and pharmacovigilance studies are critical to developing long-term evidence to provide assurances on efficacy as well as safety. These adalimumab biosimilars, and many more in the pipeline, will offer exciting opportunities in improving treatment access and increasing treatment options worldwide.
